# Editorial: Deep subsurface microbiology and energetics

**DOI:** 10.3389/fmicb.2025.1611707

**Published:** 2025-05-07

**Authors:** Alberto Robador, William J. Brazelton, James Andrew Bradley

**Affiliations:** ^1^Department of Biological Sciences, University of Southern California, Los Angeles, CA, United States; ^2^School of Biological Sciences, The University of Utah, Salt Lake City, UT, United States; ^3^Mediterranean Institute of Oceanography, Marseille, France

**Keywords:** deep biosphere, microbial energetics, redox zonation, energy-limiting ecosystems, subsurface biogeochemistry

## Introduction

Deep subsurface environments host vast and dynamic ecosystems that challenge conventional paradigms of the rules of microbial life. At the same time, these unusual ecosystems are integral to Earth's biogeochemical cycles. Because these environments are shaped by geological processes and often constrained by extreme energetic limitations, life in the deep subsurface is governed by complex interactions among redox chemistry, spatial structure, and long-term adaptation to energy scarcity. The collection of articles presented in this Research Topic, “*Deep subsurface microbiology and energetics,”* provides an interdisciplinary perspective through which to examine the metabolic potential, ecological organization, and functional resilience of microorganisms inhabiting these remote environments.

## Redox zonation and microbial niche partitioning

Microbial metabolism in the deep subsurface is tightly coupled to redox gradients established by the interactions of water, rock and biology. These gradients define not only the thermodynamic constraints for energy conservation but also the spatial partitioning of microbial niches.

In hyperalkaline fluids derived from serpentinization in the Samail Ophiolite, Howells et al. reveal that formate, rather than hydrogen or acetate, provides the most favorable energy source for methanogenesis under CO_2_-limiting conditions. This finding highlights the selective pressure exerted by fluid geochemistry on microbial metabolic strategies with the unusual example of CO_2_ as the limiting substrate. In a related system, Twing et al. investigate microbial communities within altered ultramafic rocks from the Coast Range Ophiolite. Their results underscore the distinction between rock-hosted and fluid-associated microbial assemblages, with implications for potential spatial isolation and endemism of deep biosphere lineages.

Sedimentological and fracture-hosted systems offer further insights into the persistence of microbial metabolic potential over time. In the sediments of Lake Cadagno, Rodriguez et al. document the preservation of core metabolic functions across millennia, despite taxonomic shifts in sediment microbial communities. Similarly, Acciardo et al. report stable groundwater microbial and hydrochemical compositions across an Alpine tunnel system, in spite of notable temporary variations corresponding to seismic events. These studies collectively suggest that functional redundancy and environmental buffering contribute to the resilience of deep subsurface microbiomes.

## Strategies for survival in energy-limited settings

Adaptation to energy scarcity is a hallmark of deep subsurface systems. Survival in the energy-limited subsurface depends not only metabolic versatility but also regulatory and genomic mechanisms attuned to long-term energy deprivation and survival over long timescales.

Sebastian et al. address this challenge through an experimental evolution framework, demonstrating that *Halomonas* strains isolated from North Pond crustal fluids rapidly lose the genetic capacity for survival in carbon-depleted media when exposed to nutrient-rich laboratory conditions. Their findings point to specific gene products, including lipase-like proteins, as key mediators of nutrient acquisition under oligotrophic conditions.

Complementing this genomic perspective, Feyhl-Buska et al. use microcalorimetry to directly quantify energy metabolism in groundwater samples spanning a range of redox conditions, revealing nutrient limitation in continental subsurface communities. The results provide empirical constraints on microbial power output and underscore the influence of both thermodynamic and ecological parameters on community-level metabolism.

## Beyond classical metabolism: novel energetic pathways and functional innovation

Emerging theoretical frameworks and cultivation-based discoveries continue to expand the known boundaries of microbial energetics.

Karnachuk et al. describe the isolation of two novel thermophilic bacterial genera from deep aquifers in Western Siberia. These members of the *Limnochordia* lineage possess the capacity for carbon monoxide and hydrogen oxidation, autotrophic carbon fixation, and aerobic respiration. Their physiological versatility illustrates the metabolic plasticity that may underpin survival in thermally and chemically extreme environments.

A conceptual advance is presented by Marshall, who proposes the existence of Electromicrobiological Concentration Cells (EMCCs). These hypothetical systems rely on conductive matrices to bridge spatially distinct redox reactions, allowing microorganisms to exploit electrochemical disequilibria that would otherwise be inaccessible. The EMCC framework challenges traditional assumptions about the spatial coherence required for microbial energy harvesting and may have relevance for life in both deep terrestrial and extraterrestrial settings.

## Coupled biogeochemical cycling and ecosystem regulation

The influence of subsurface microbial communities extends to the regulation of key geochemical processes, including methane production and oxidation, nutrient turnover, and mineral transformation.

Coon et al. demonstrate that the directionality of methane cycling in coastal sediments is modulated by hydrogen availability. In organic-poor settings, syntrophic interactions between fermenters and methanogens or sulfate reducers maintain low hydrogen concentrations, promoting fermentation and anaerobic methane oxidation. Conversely, elevated hydrogen levels in organic-rich sediments destabilize these interactions, favoring methanogenesis.

In the context of engineered subsurface environments, Cheng et al. investigate microbial community dynamics in oil reservoir cores subjected to nutrient amendments. Their findings indicate that nutrient limitation supports greater community diversity and network stability, whereas nutrient enrichment promotes dominance by functionally narrow taxa and reduces ecological resilience.

This theme is extended by Mura et al., who simulate dihydrogen (H_2_) co-injection in a natural gas–bearing, low-salinity deep aquifer under controlled laboratory conditions. Using a high-pressure reactor system with native rock and fluid phases, they show that microbial activity leads to sequential reduction processes, including sulfate reduction, acetogenesis, and ultimately methanogenesis, with the latter accounting for the majority of H_2_ consumption. While microbial H_2_ uptake remained limited—likely due to nutrient depletion—the work reveals that aquifers with low salinity and restricted electron acceptor availability may provide relatively stable reservoirs for H_2_ storage. This study further emphasizes the need for site-specific microbial and geochemical assessment when evaluating the feasibility of underground H_2_ storage.

Further advancing the discussion on trace element-driven microbial processes, Montoya and Escobar-Briones explore the role of cobalt and vitamin B_12_ (cobalamin) in structuring microbial and metazoan communities associated with ferromanganese crusts and polymetallic nodules. These cobalt-rich habitats host diverse microbial assemblages, including cobalamin-producing prokaryotes such as Thaumarchaeota and Proteobacteria, which support cobalamin-auxotrophic eukaryotes via trophic and symbiotic interactions. The authors argue that the abundance of cobalt in these deep-sea mineral systems may create micronutrient “hotspots” where metabolic interdependencies become intensified. This framework posits cobalt and cobalamin as pivotal drivers of deep-sea microbial ecology and proposes that these mineral-rich habitats offer valuable models for studying micronutrient-based ecological networks and their vulnerability to anthropogenic disturbance.

## Integrating the deep subsurface biosphere into planetary systems thinking

A broader systems perspective is articulated by Robador, who conceptualizes the oceanic crustal biosphere as a globally distributed, low-energy biogeochemical reactor. This framework situates subseafloor microbial processes within Earth's carbon cycle and emphasizes the significance of subsurface ecosystems in global redox balance. By drawing parallels between deep biosphere functions and potential analogs on other planetary bodies, the review reinforces the role of subsurface microbiology in astrobiology and Earth system science.

## Concluding remarks

The contributions compiled in this Research Topic underscore the multifaceted nature of deep subsurface microbial life. From theoretical advances and empirical discoveries to environmental and engineered system analyses, these studies highlight the central role of microbial subsurface communities in mediating geochemical processes under extreme conditions. As exploration of subsurface environments continues—on Earth and beyond—the principles of microbial energetics, adaptation, and resilience described herein will serve as a foundation for understanding life in one of its most enigmatic domains.

This Research Topic is in memory of Jan Amend, whose work and ideas, mentorship, enthusiasm and leadership not only transformed the field of deep subsurface microbiology but also inspired and guided a generation of scientists that have continued to advance this field.

